# Magnetoresistive Properties of Nanocomposites Based on Ferrite Nanoparticles and Polythiophene

**DOI:** 10.3390/nano13050879

**Published:** 2023-02-26

**Authors:** Roma Wirecka, Krzysztof Maćkosz, Antoni Żywczak, Mateusz Marek Marzec, Szczepan Zapotoczny, Andrzej Bernasik

**Affiliations:** 1 Faculty of Physics and Applied Computer Science, AGH University of Science and Technology, al. Adama Mickiewicza 30, 30-059 Krakow, Poland; 2 Academic Centre for Materials and Nanotechnology, AGH University of Science and Technology, al. Adama Mickiewicza 30, 30-059 Krakow, Poland; 3 Empa-Swiss Federal Laboratories for Materials Science and Technology, Laboratory for Mechanics of Materials and Nanostructures, Feuerwerkerstrasse 39, CH-3602 Thun, Switzerland; 4 Faculty of Chemistry, Jagiellonian University, ul. Gronostajowa 2, 30-387 Krakow, Poland

**Keywords:** magnetic nanoparticles, magnetoresistance, magnetic nanocomposites

## Abstract

In the presented study, we have synthesized six nanocomposites based on various magnetic nanoparticles and a conducting polymer, poly(3-hexylthiophene-2,5-diyl) (P3HT). Nanoparticles were either coated with squalene and dodecanoic acid or with P3HT. The cores of the nanoparticles were made of one of three different ferrites: nickel ferrite, cobalt ferrite, or magnetite. All synthesized nanoparticles had average diameters below 10 nm, with magnetic saturation at 300 K varying between 20 to 80 emu/g, depending on the used material. Different magnetic fillers allowed for exploring their impact on the conducting properties of the materials, and most importantly, allowed for studying the influence of the shell on the final electromagnetic properties of the nanocomposite. The conduction mechanism was well defined with the help of the variable range hopping model, and a possible mechanism of electrical conduction was proposed. Finally, the observed negative magnetoresistance of up to 5.5% at 180 K, and up to 1.6% at room temperature, was measured and discussed. Thoroughly described results show the role of the interface in the complex materials, as well as clarify room for improvement of the well-known magnetoelectric materials.

## 1. Introduction

Having both electrical properties and magnetic response, with addition of possible flexibility and ease of synthesis, nanocomposites based on conducting polymers and magnetic nanoparticles are of high interest both in the scientific and commercial worlds [1,2]. Continuous studies aiming at a better understanding of their electrical properties, the influence of the filler, and the interface between components, on the final properties of the nanocomposite materials resulted in numerous reports showing new methods for their synthesis and potential applications [3,4,5,6,7,8].

Among many interesting and adjustable properties of such composite materials, the alteration of their properties under the influence of the magnetic field (magnetic field effects—MFE) is of high interest. The influence of the magnetic field was observed to have an impact on the photocurrent (magneto-photocurrent) [9,10], electroluminescence (magneto-electroluminescence) [11,12], electrical current (magneto-electrical current, magnetoresistance, magnetoconductance) [13,14], and other properties [15]. All those properties mainly originate from three phenomena: carrier recombination, exciton dissociation, and electric polarization. As the external magnetic field introduces coherent and incoherent spin precessions, the spin of the electron may be affected. As a result, it can remain unchanged or it can alter its orientation, which leads to positive or negative MFEs, respectively [16].

Among other MFEs, one that is constantly drawing attention to the polymer-magnetic nanoparticle composites is magnetoresistance (MR). It is described as the alteration of the electrical resistivity (R) of the material in the external magnetic field (H).

(1) $$\%\mathrm{MR}=\frac{R_{H}-R_{0}}{R_{0}}\times 100\%$$

The positive MR is seen as the increase, while the negative MR results in the decrease of the resistivity in the external magnetic field. The use of magnetic semiconducting nanoparticles allows for tuning the conductivity of the material [17,18]. Thanks to the magnetic properties of the filler, the magnetic response can be modified, and the MR can be amplified [19].

This study presents the synthesis of six different core–shell nanoparticles which are later suspended in the electrically conductive polymer matrix to obtain nanocomposites with magnetoelectric properties. The shell of the nanoparticle, made of either insulating material (squalene and dodecanoic acid) or conducting material (poly(3-hexylthiophene-2,5-diyl)), covers a magnetic core made of one of three different ferrites. The morphology and magnetic properties of the nanoparticles are thoroughly analyzed to help understand the differences between the obtained nanoparticles. Finally, the electrical and magnetoresistive properties of thin composite films made of nanoparticles suspended in P3HT are carefully studied to establish the influence of the shell-covering nanoparticle on the conductivity mechanism.

## 2. Materials and Methods

### 2.1. Materials

Fe(acac)_3_ (acac = acetylacetonate) (97%), Ni(acac)_2_ (97%), Co(acac)_2_ (97%), dibenzyl ether (98%), squalene (95%), dodecanoic acid (98%), and anhydrous dichlorobenzene were purchased from Sigma Aldrich. Poly(3-hexylthiophene-2,5-diyl) (P3HT) M_w_ = 51,000 u and over 90% regioregularity was purchased from Rieke Metals. All reagents were used as received.

### 2.2. Synthesis of Nanoparticles Capped with Squalene

The synthesis of the Fe_3_O_4_ nanoparticles (Fe(Sq)) was based on the thermal decomposition of the acetylacetonates in a controlled argon gas atmosphere, thoroughly described in our previous work [20]. The synthesis of cobalt (Co(Sq)) and nickel ferrites (Ni(Sq)) followed the same protocol, but the molar ratio of iron to dopant was chosen to be 2/1. The synthesis consisted of three stages: dissolving of the solution (at 80 °C for one hour), degassing (at 200 °C for one hour), and the formation of nanoparticles from decomposed metal acetylacetonates (at 280 °C for one hour). After this time the content of the flask is left overnight to cool down and then cleaned with acetone by centrifugation (10 min, 10,000 rpm) at least three times. Afterward, the nanoparticles are suspended in the dichlorobenzene and stored in the dark at 4 °C.

### 2.3. Synthesis of Nanoparticles Capped with P3HT

The synthesis of magnetite nanoparticles capped with P3HT (Fe(P3HT)) was already described in our previous work [20]. The synthesis of nickel (Ni(P3HT)) or cobalt (Co(P3HT)) ferrite follows the same procedure with the molar ratio of iron to the chosen dopant being 2/1. Briefly, the synthesis consists of three steps. Firstly, a flask containing P3HT and dibenzyl ether is kept at 100 °C for 12 h in dark to assure good dissolution of the polymer. Then, the solution is transferred to the pre-heated (200 °C) flask containing dibenzyl ether and the proper amount of metal acetylacetonate and kept for 90 min. The final stage, in which the decomposition of the acetylacetone and nanoparticles’ formation takes place, lasts 60 min at 280 °C. After the synthesis, the mixture is kept in argon gas for 12 h to cool down, then is cleaned with acetone by centrifugation (10 min, 10,000 rpm). Cleaned nanoparticles are suspended in the dichlorobenzene and kept in the dark at 4 °C.

### 2.4. Nanocomposite Preparation

Nanoparticles obtained following the previously described procedures (NP(Sq) for nanoparticles capped with squalene and NP(P3HT) for nanoparticles covered with P3HT) were suspended in P3HT dissolved in dichlorobenzene. The concentration of polymer was set to 14 mg/mL and nanoparticles to 10 mg/mL. The solutions were put in the ultrasonic bath for one hour to ensure good distribution of the nanoparticles in the polymer solution. Six different prepared solutions were further used to obtain nanocomposite films.

### 2.5. Vibrating Sample Magnetometry

For the magnetic characterization of obtained nanoparticles, a vibrating sample magnetometer (VSM), type 7407 Lake Shore Cryotronics, Inc., (Westerville, OH, USA) was chosen. Before the measurement, the nanoparticles were dried, and as a powder transferred to the apparatus in a Teflon vessel. All measurements were conducted at three temperatures: low (80 or 100 K), nearly room temperature (290 or 300 K), and high (440 K), in an external magnetic field ranging from –1500 mT to 1500 mT.

### 2.6. Conductivity Measurements of Nanocomposites

Substrates for the conductivity measurements were custom made. They consisted of two 100 nm thick gold electrodes evaporated on top of the SiO_2_ wafer/substrate. The distance between electrodes was set to 75 µm.

Nanocomposite solutions were drop cast on the described substrates in the argon-filled glovebox (O_2_ ≤ 0.1 ppm and H_2_O ≤ 0.1 ppm). The samples were annealed at 130 °C for 15 min and left overnight to cool down. Afterward, the contact pads were cleaned with a cleanroom swab dipped in dichlorobenzene. Samples were transferred in the isolated sample holder to the VSM apparatus equipped with a conductivity measurement holder. The continuous nitrogen gas flow in the apparatus ensured the elimination of the oxygen and humidity in the vicinity of the sample. Conductivity measurements were carried out using the two-point method with a Keithley 2400 source meter. The distance between the electrodes was set to 75 µm. The R(T) characteristics were collected at different temperatures ranging from 200 K to 400 K.

### 2.7. Conductivity Measurements of Nanocomposites in the External Magnetic Field

Samples were prepared in the same manner as for R(T) measurements. The external magnetic field was applied parallel to the sample’s surface. The magnetic field was applied in the sequence starting at 0 mT, then increasing to 1500 mT, and then changing to −1500 mT, and finishing at 0 mT with the 10 mT/s step and 5 V applied to the sample to measure the magnetoconductivity of the sample. The same measurements were conducted in the temperature regime ranging from 300 K to 200 K, all with the same sequence.

## 3. Results

### 3.1. Morphology of the Nanoparticles

**Figure 1:**
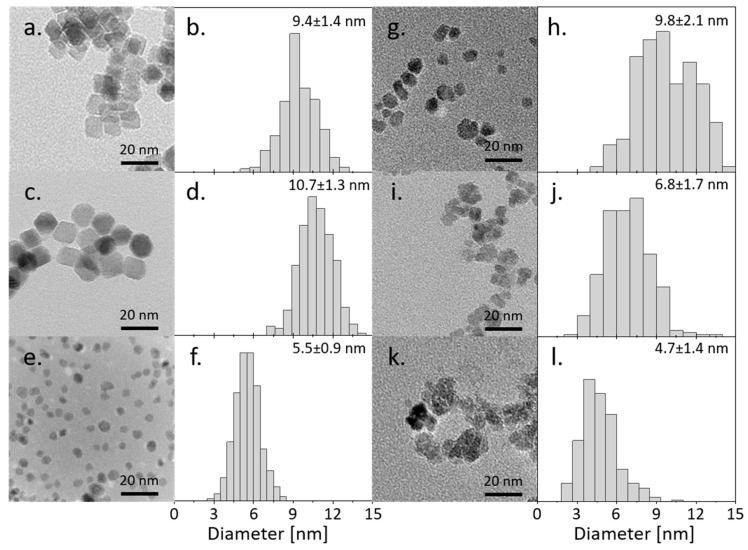
TEM micrograph with corresponding histograms of synthesized nanoparticles: (**a**,**b**) Fe(Sq); (**c**,**d**) Co(Sq); (**e**,**f**) Ni(Sq); (**g**,**h**) Fe(P3HT); (**i**,**j**) Co(P3HT); (**k**,**l**) Ni(P3HT).

The morphology with the corresponding histograms of all six types of obtained nanoparticles is presented in Figure 1**.** The Fe(Sq) and Co(Sq) samples are characterized by regular cubic and hexagonal shapes with ~10 nm diameter, while Fe(P3HT) samples have the same mean diameter (~10 nm) but their dispersity of size is higher. The Co(P3HT) particles are slightly smaller (~7 nm) and irregular, similar to Fe(P3HT). The most regular shapes, hence least defected structures, were obtained from the pure Fe_3_O_4_ and nanoparticles doped with cobalt in the presence of surface agents—squalene and dodecanoic acid. There is a visible distinction between nanoparticles synthesized directly in the polymer matrix and the surface agent—nanoparticles in the presence of polymer become more irregular and smaller. The aggregation of nickel and cobalt ferrite nanoparticles is similar, but pure Fe_3_O_4_ nanoparticles synthesized in the presence of polymer are not aggregated at all. A possible explanation is that the magnetic interactions between the particles are much smaller in comparison to the Fe(Sq) or Co(Sq), which can be established during the VSM measurement. Secondly, there is a possibility the polymer coverage is stabilizing small nanoparticles. The dispersity of sizes between NP(Sq) and NP(P3HT) is similar in two out of three types of nanoparticles, which is surprising and shows that the use of size- and shape-controlling mediums during the synthesis, such as squalene and dodecanoic acid, has little effect on the distribution of sizes, but affects the regularity of obtained shapes.

### 3.2. Magnetic Characterization

**Figure 2:**
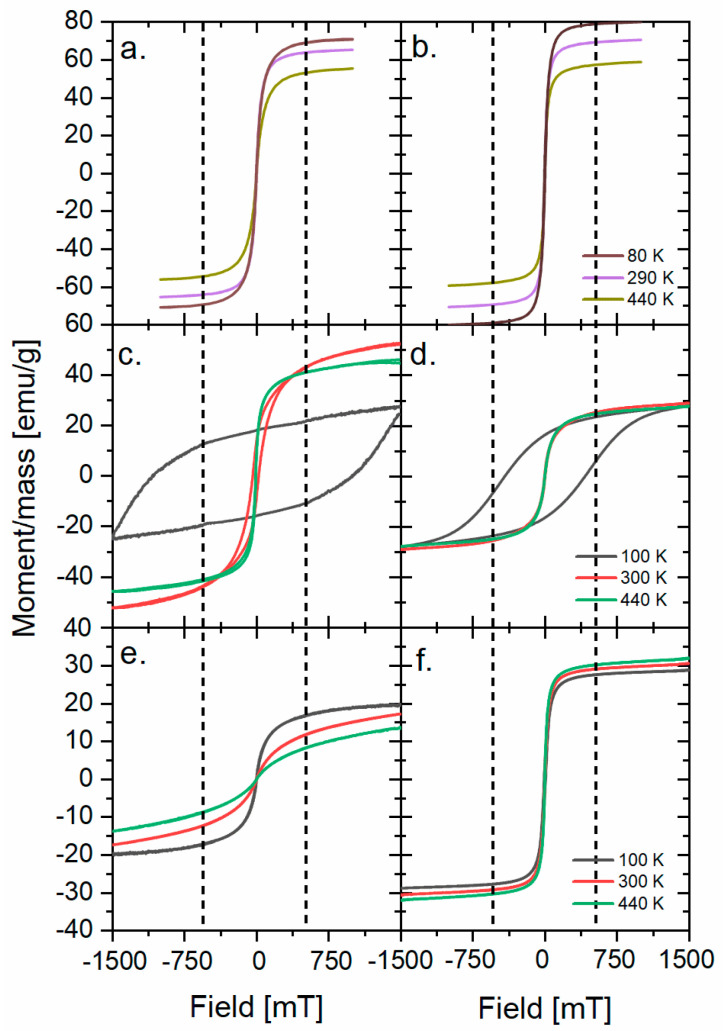
M(H) loops for (**a**) Fe(Sq), (**b**) Fe(P3HT), (**c**) Co(Sq), (**d**) Co(P3HT), (**e**) Ni(Sq), and (**f**) Ni(P3HT). The dotted lines show the field value for which the magnetization saturation for 300 K is observed.

The magnetic hysteresis loops of as-received nanoparticles at three different temperatures are presented in Figure 2. The saturation magnetization (M_s_) of all synthesized nanoparticles with corresponding bulk values taken from the literature are presented in Table 1. The M_s_ is a characteristic value of the field that aligns all of the magnetic moments in the sample. The comparison of alterations of values between the bulk and the nanoparticles allows making some assumptions about the magnetic dead layer (MDL) [19] and the influence of the synthesis on the magnetic properties and structure. The largest difference between the bulk value and the M_s_ of the nanoparticle is observed for Co(P3HT) and Ni(Sq) nanoparticles, which is probably caused by the structural distortions present at the surface of the nanoparticles in the samples. Additionally, almost no difference of M_s_ is observed for magnetite nanoparticles, showing that the magnetic properties are similar regardless of the less regular size of Fe(P3HT) and different outer layers. The M_s_ of nickel nanoparticles is four times smaller than magnetite (and two times smaller than cobalt ferrite nanoparticles), which is a result of the highest proportion between the surface and the core. Since the surface may be treated as a defected structure, the MDL will have the biggest impact on the magnetic properties when the nanoparticles’ size is decreasing. The results for Co(P3HT) nanoparticles support this hypothesis: Co(Sq)’s M_s_ and the nanoparticle’s diameter are two times bigger than those of Co(P3HT). Worth pointing out is the lack of (or very low) coercivity in almost all samples (Co(Sq) in 100 K being one exception), which shows that the addition of polymer to the nanoparticles does not have a negative, insulating impact on the magnetic properties of the composite, which was observed for a similar P(VDF-H FP)/Cobalt ferrite nanocomposite [21].

Next, the electrical properties of nanocomposites based on the nanoparticles described above were checked. The materials were studied without and with the magnetic field applied during the conductivity measurements.

### 3.3. Electric Properties

**Figure 3:**
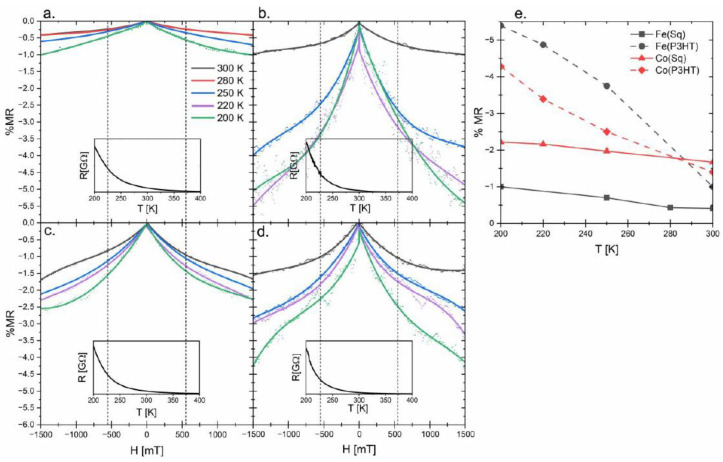
Graphs presenting changes in the magnetoresistance: (**a**) Fe(Sq), (**b**) Fe(P3HT), (**c**) Co(Sq), and (**d**) Co(P3HT); the lines present in the graphs are to guide the over the results; the insets of the R(H) characteristics prove semiconducting properties of obtained materials; (**e**) is a summary of the MR values in the 1500 mT at different temperatures.

To determine the electrical conductivity mechanism of obtained nanocomposites, the resistivity of the samples was measured at temperatures ranging from 200 to 400 K. Because the resistivity is decreasing with the increase of temperature, the nanocomposites are typical semiconducting materials with a negative temperature coefficient (insets in Figure 3). Such results suggest a thermally activated conduction mechanism [25] in which the charge carriers are tunneling or hopping between nanoparticles and P3HT.

The conductivity of polymers and their composites strongly depends on the structural order of the system [26]. One of the elements affecting it is the disorder of the polymer chains, which may be influenced by the addition of nanoparticles. The disordered state can be defined as the conductivity ratio measured at different temperatures, or by the Mott temperature calculated from the Mott Variable range hopping conductivity model (VRH) [27]. To express the value of the composite’s disorder, the ratio of the resistance values at 200 K and 300 K was calculated (Table 2). For the pure P3HT, it was not possible to measure resistance below 300 K in our measuring setup, so the disorder was not analyzed, hence in the discussion only synthesized composites are included. 

The conductivities of nanoparticles covered with squalene and dodecanoic acid were measured. The results showed that the resistivity of the samples is so high that it can be concluded that nanoparticles are insulators. For the nanoparticles covered with P3HT, the conductivity was measurable [20]. Only after creating nanocomposites, was it possible to observe electrical conductivity for materials based on both NP(Sq) and NP(P3HT). Since conductivity was measured for all synthesized nanocomposites, the significant influence of the nanoparticles in charge carrier transport is proven. The composites in which nanoparticles were covered with squalene show a more than two times smaller disorder ratio than those in which nanoparticles were coated with polymer. Additionally, it can be observed, that with the change in type of ferrite, the ratio increases from 11.1 for Fe(Sq) to 17.8 for Ni(Sq) and from 28.5 for Fe(P3HT) to 44.7 for Ni(P3HT). These results show that the addition of nanoparticles affects the conducting behavior of the samples in a consistent manner. One probable explanation of the disorder difference is that squalene insulates nanoparticles, which causes only small alterations of the polymer matrix conduction paths. On the other hand, when the composite consists of the polymer and NP(P3HT), the inclusion of chains covering the magnetic nanoparticle in the polymer matrix causes additional distortions and interactions between polymer chains, which greatly increases the disorder of the conduction paths. Furthermore, in the materials based on NP(P3HT), the highly spin-polarized charge carriers injected from the nanoparticles may be transferred to the polymer chain and influence the conductivity of the polymer matrix. In the case of NP(Sq), the barrier may be too high to inject any charge carrier into the polymer matrix [28]. 

To further study the conduction mechanism of the synthesized materials, the VRH model is applied. Following Guo et al. [29], Equation (2) was used to determine it.

(2) $$ln\left(\frac{1}{R}\right)=ln\left(\frac{1}{R_{0}}\right)-{\left(\frac{T_{0}}{T}\right)}^{\frac{1}{1+n}}n=1,\;2,\;3$$

In Equation (2), *R* is the resistivity of the sample, *R*_0_ is the resistivity of the sample at the infinitely low temperature, *T* is the temperature (K), and *n* is the constant <1,3>, which reflects the dimension of the system. Mott temperature (*T*_0_) is related to the decay length of the localized wave function of the charge carriers and the DOS at the Fermi level and is also known as a hopping barrier [30]. The results of the calculations are summarized in Table 2. Since the *T*_0_ is correlated with the disorder of the polymer [31], the results should also reflect the data for the resistivity ratio. Such correlations are observed for four out of six studied composites. For magnetite and cobalt ferrite nanoparticles, both the resistivity ratio and *T*_0_ increase for samples covered with P3HT. This correlation and the fact that samples follow the function trend very well suggest a quasi-3D VRH electrical conduction mechanism for the four measured samples. We believe that the hopping of the carriers may occur between P3HT covering NP(P3HT) and the polymer matrix, and at the same time, the transport may occur along the polymer backbone [29,32]. On the other hand, when nanoparticles are covered with the P3HT, the polymeric shell may affect the polymer chains present in the sample. Additionally, the magnetic core of the nanoparticles stabilizes the spin of the charge carriers and lowers the probability of scattering. Since the magnetic fringe field decreases as the third power of the diameter, if the nanoparticle is not covered with any insulating layer, the influence of such magnetic core is higher, which also leads to lowering the scattering probability.

### 3.4. Magnetoresistive Properties

The magnetoresistance of four out of six synthesized materials is presented in Figure 3. In the case of pure P3HT and nickel ferrite nanoparticles, no change in the resistivity was observed. Since no response was observed for pure polymer in the VSM measurement, no response to the external magnetic field was expected for these samples. For the nickel-doped nanoparticles, the VSM measurement suggested MDL covering nanoparticles, which may influence the spin orientation of the charges leaving the core of the nanoparticle [19]. Additionally, the charge carrier transport through the polymer matrix is limited by hopping between less-disordered regions [33]. Knowing that it is highly probable that these two effects occur in the materials synthesized with nickel-containing nanoparticles, no MR was expected. Nanocomposites based on magnetite and cobalt ferrite, show different results. For each of the thin films, the resistance of the material is decreasing with the increase of the applied magnetic field (Figure 3e). This is the result of the lowered scattering of the charge carriers between polymer chains and/or nanoparticles present in the material [34]. Even though saturation magnetization is observed for all samples at all temperatures (Figure 2), no saturation of the magnetoresistivity is observed. The hyperfine interactions and spin-orbit splitting have the most significant influence on MR in the field strength up to 500 mT; thus these two phenomena do not explain the conductivity response to the increase in the magnetic field. Worth noting is the fringe field coming from the core of magnetic nanoparticles. In the presented system, nanoparticles are distributed throughout the polymer matrix, which possibly influences the MR in high-strength fields. The described magnetoresistance results are summarized in Figure 3e. For NP(P3HT), the comparison of the results, obtained at room and lower temperatures, shows that even a small change of temperature allows for obtaining much better results. At 200 K, which was the lowest measured temperature, the MR in 1500 mT for Fe(P3HT) reaches almost 5.5%, which is 5.5 times better than observed in the composite containing NP(Sq). It is clearly visible that the composites based on the NP(Sq) show a smaller change of the conductance even in the high-strength fields, and it does not change with the alteration of the temperature. These distinctions between the two types of composites, and the lack of saturation of MR, may be explained by the forward interference model proposed by Nguyen, Spivan, and Shlovskii (the NSS model) [35]. In this model, the conductivity of a material is a sum of all possible conduction paths, so when NP(Sq) is used as a filler, it does not add much to the conductivity, but when nanoparticles are covered with the conductive shell, the enhancement should occur. If we take into consideration the T_0_ (which is often taken as a parameter connected to the charge carrier scattering probability [27]) and the fact that at lower temperatures the chance of scattering is lower, then the impact of the shell may play a significant role in the number of possible conduction paths and their interference, which is visible in our results. Additionally, in this model, no saturation of magnetoresistance for material is observed even in high-strength fields. 

## 4. Conclusions

In conclusion, we have created six different nanocomposites, based on magnetic nanoparticles, covered with two types of shells (insulating and conducting), and an electrically conductive polymer matrix. Electrical conductivity measurements have proven a significant influence of the nanoparticles on the charge carrier transport. Results suggest a quasi-3D VRH electrical conduction mechanism for nanocomposites in which magnetite, cobalt, or nickel ferrite nanoparticles were used as fillers. Moreover, we have shown that the choice of shell-covering for the magnetic nanoparticle has a major impact on both the conduction paths in the nanocomposites and the probability of charge carrier scattering. Furthermore, we have shown higher negative magnetoresistance for nanocomposites based on nanoparticles covered with a conductive polymeric shell. We have observed the relationship between the saturation magnetization of nanoparticles and the magnetoresistance change. The highest magnetoresistance variation, of up to 5.5% at a magnetic field of 1500 mT, was measured for the nanocomposite based on the magnetite nanoparticles covered with a conductive shell. As a takeaway summary, our nanocomposites which combine electrical conductivity with a magnetic response can be used as easily made thin sensors of magnetic fields or as new scalable materials for organic spintronic applications.

## Figures and Tables

**Table 1:** Summary of sizes and saturation magnetization in 300 K of all samples.

Sample	Diameter [nm]	Experimental M_s_ for NPs [emu/g]	M_s_ for Bulk at 300 K [emu/g]
Fe(Sq)	9.4 ± 1.4	65.5	96.0 [22]
Fe(P3HT)	9.8 ± 2.1	70.0	
Co(Sq)	10.7 ± 1.3	52.7	80.8 [23]
Co(P3HT)	6.8 ± 1.7	28.9	
Ni(Sq)	5.5 ± 0.9	16.9	55.0 [24]
Ni(P3HT)	4.7 ± 1.4	30.6	

**Table 2:** Disorder ratio for different types of synthesized nanocomposites with calculated resistance at low temperature and Mott’s temperatures (T_0_).

Nanocomposite Sample	Resistivity Ratio R_200K_/R_300K_	R_0_ [Ohm]	T_0_ 10^7^ [K]
Fe(Sq)	11.1	8.8 × 10^−4^	9.22
Fe(P3HT)	28.5	2.0 × 10^−11^	94.37
Co(Sq)	14.6	2.8 × 10^−5^	11.75
Co(P3HT)	33.6	2.5 × 10^−7^	29.15
Ni(Sq)	17.8	1.7 × 10^−5^	45.32
Ni(P3HT)	44.7	6.0 × 10^−7^	32.47

## Data Availability

The data presented in this study are available on request from the corresponding author.
